# Utility of totally implantable venous access ports in patients with breast cancer

**DOI:** 10.1111/tbj.13595

**Published:** 2019-09-20

**Authors:** Peng Zhang, Jun Du, Changsheng Fan, Xueli Mo, Jie Dong, Zhenhua Fan, Qikang Zhao

**Affiliations:** ^1^ Department of Breast Diseases Peking University Shougang Hospital Beijing China

In recent years, totally implantable venous access port (TIVAP) is increasingly applied in the patients’ chemotherapy which was usually involved in cancer treatment.^1^ It is necessary for some patients with breast cancer to undergo chemotherapy more than 6 months. In addition, some drugs are toxic to the veins particularly peripheral veins.^2^ So the implantation of a TIVAP is required as its advantage of easy access of central veins.^3^ It was reported that there were significant less complications in TIVAPs than what in other accesses for chemotherapy and it could be used in entire treatment cycle safely.^4^ Generally, in TIVAPs implantation, we use the basilic vein, subclavian vein, external jugular vein, or the internal jugular vein (IJV) as puncture sites. Since some patients with breast cancer may receive ipsilateral radiation treatment, the veins and chest wall of the contralateral side are the suitable choices for TIVAP implantation.^5^ From January 2013, the TIVAP implantation with IJV or subclavian vein as puncture site has been launched in our department. The chamber of the port has been placed into the chest wall of the healthy side, and the catheter has been introduced to the superior vena cava (SVC). In this study, we would like to investigate and analyze the key procedures and complications of TIVAPs implanted by blind puncture or preoperative ultrasonic marker. We aim to assess the safety of TIVAP implantation via blind puncture and evaluate the feasibility of port system and optimal time of chemotherapeutic drugs infusion for breast cancer.

We collected 110 patients with breast cancer for this study. All patients received chemotherapy after TIVAPs implantation. Between January 2013 and December 2017, 110 patients with breast cancer underwent TIVAPs implantation by blind puncture or preoperative ultrasonic marker point in our department. The operation time was measured from the time when the patient received local anesthesia to the time when the patient left the operation table. Early complications were defined as the complications occurred in 3 days after TIVAPs introduction, and late complications were defined as the complications arose after 3 days after operation. The chest x‐ray examination was arranged for location confirmation of port implantation immediately after operation. The suitable position of the tip of infusion set was the joint area of lower third of the SVC. The achievement ratio and operation duration by two means were shown in Table 1.

**Table 1:** Comparison of achievement ratio and operation duration by different puncture methods and sites

Implantation method/site	No.	Achievement ratio (%, n/N)	Duration of operation (m)
Blind puncture	98		
Right internal jugular	57	100.00 (57/57)*	41.3*
Left internal jugular	20	100.00 (20/20)	43.2
Right subclavian	10	100.00 (10/10)*	39.5*
Left subclavian	11	100.00 (11/11)	40.7
Preoperative ultrasonic marker	12		
Right internal jugular	4	100.00 (4/4)*	43.4*
Left internal jugular	2	100.00 (2/2)	45.3
Right subclavian	2	100.00 (2/2)*	42.3*
Left subclavian	4	100.00 (4/4)	44.8

* *P* > .05

The median age of our patient cohort was 52 (range: 34‐72 years). All TIVAPs were implanted for cancer chemotherapy. The mean operation time of TIVAP placement was 42.5 minutes (33‐75 minutes). The locations of TIVAPs on the right upper chest walls were observed in 73 cases (66.36%) and 37 cases were found on the left upper chest walls (33.64%). The inserted vein was the IJV in 83 cases (75.45%) and the subclavian vein chosen as puncture site was observed in 27 cases (24.55%). All TIVAPs were successfully implanted in our department. The technique of the blind puncture was applied to 98 patients (100.00% success rate) and the method of preoperative ultrasonic marker was used in the remaining patients (100.00% success rate). No significant difference was found between two groups (*P* > .05). In further analysis, mean duration of operation via blind puncture is 41.2 and 43.9 minutes via preoperative ultrasonic marker. No significant difference was observed in two methods (*P* > .05).

As shown in Table 2, in our investigation, in 98 cases by blind puncture, 10 patients were found to be involved in complications (10.20%). One patient was observed to be involved in complication in 12 cases (8.33%). It is no significant difference between two groups (*P* > .05). The overall rate of complication 10.00% was lower than which in previous reports.^4^, ^6^, ^7^ In addition, the specific time of port application is controversial. Some doctors thought that chemotherapeutic drugs were given by port system on a port implantation day appeared safe without increasing any complication.^8^ However, Narducci F et al^9^ reported that chemotherapy via port system after at least 8 days could decrease the rate of complications. In our study, seven cases were found to be involved in complications in the group of port application on TIVAP implantation day and four patients were observed to have complications in the group of port application 7 days after TIVAP implantation day. There was no significant difference between two groups (*P* > .05). Our study showed that chemotherapeutic drugs transfusion was safe on the TIVAP implantation day.

**Table 2:** Comparison of complications by two means of puncture

Complications		Blind puncture (N = 98)	Preoperative ultrasonic marker (n = 12)
Early complications	Hematoma	1	0
	Bleeding	1	0
	Cardiac arrhythmia	1	0
		3 (3.06%)	0 (0.00%)
Late complications	Infection	1	0
	Cutaneous necrosis	2	1
	Turn‐over of chamber	1	0
	Venous thrombosis	3	0
		7 (7.14%)	1 (8.33%)
Total		10 (10.20%)*	1 (8.33%)*

Note

n: total number of cases who underwent surgery via preoperative ultrasonic marker;N: total number of patients who underwent surgery by blind puncture.

* *P* > .05.

Overall, our study showed that TIVAP implantation via IJV or subclavian puncture without ultrasonic guide was safe and feasible for the breast cancer patients during adjuvant treatment and follow‐up. Chemotherapeutic drugs giving on the port implantation day was safe without an increased risk of acute and chronic complications. Particularly, in primary hospital, this technique should be widely used as its low cost and safety.
